# Spontaneous intercostal artery bleeding in a patient with alcohol‐induced liver cirrhosis

**DOI:** 10.1002/ccr3.4613

**Published:** 2021-08-16

**Authors:** Mandeep Singh Rahi, Prachi Pednekar, Gaurav Parmar, Lauren Keibel, Kulothungan Gunasekaran, Kwesi Amoah, Christopher Winterbottom

**Affiliations:** ^1^ Division of Pulmonary Diseases and Critical Care Medicine Yale‐New Haven Health Bridgeport Hospital Bridgeport CT USA; ^2^ Department of Internal Medicine Yale‐New Haven Health Bridgeport Hospital Bridgeport CT USA; ^3^ Department of Radiology Yale‐New Haven Health Bridgeport Hospital Bridgeport CT USA

**Keywords:** alcohol liver disease, cirrhosis, embolization, hemorrhagic shock, intercostal artery

## Abstract

Spontaneous intercostal artery bleeding is a rare disease seen in cirrhosis and can present with hemodynamically significant blood loss anemia, hypotension, and shock. Transcatheter arterial embolization is an effective treatment for severe cases.

## Graphical abstract

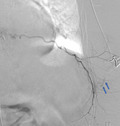

## 1 CASE PRESENTATION

**FIGURE 1:**
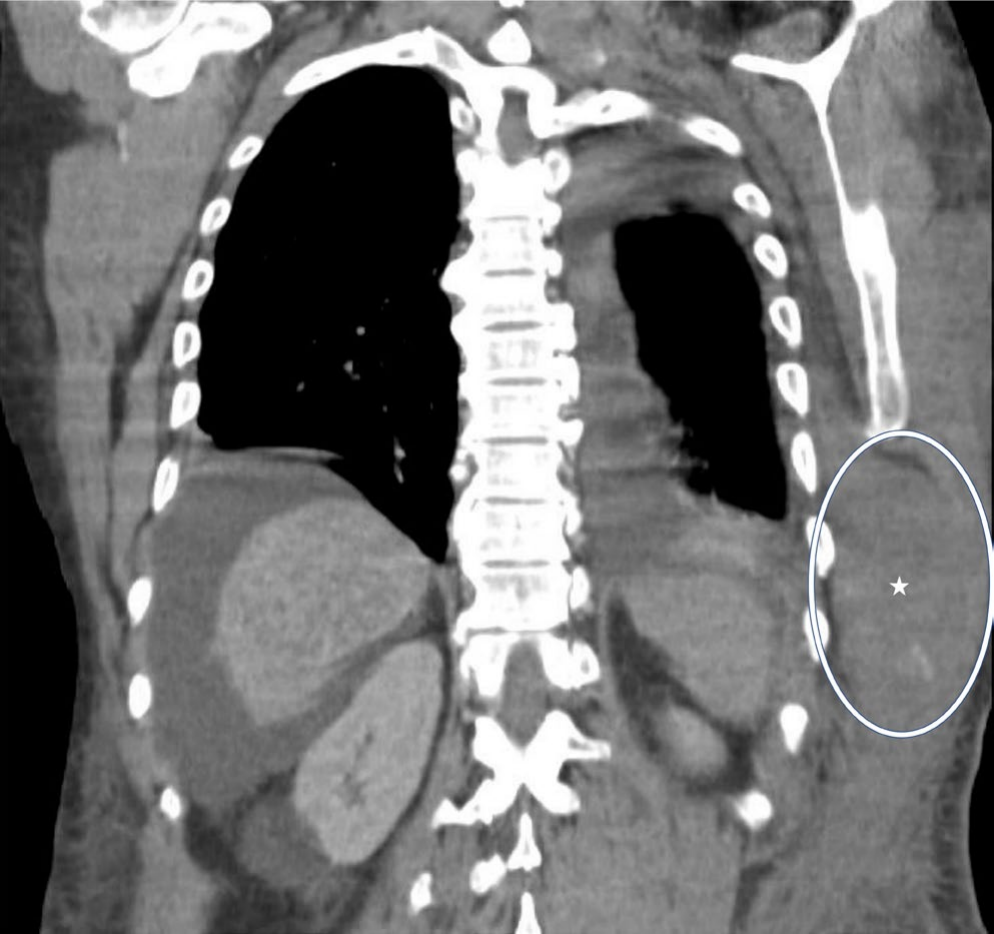
A computed tomography of the chest and abdomen in the coronal view showing a large intramuscular hematoma within the left lateral chest wall and extending to the upper abdomen, denoted by a star and a circle

**FIGURE 2:**
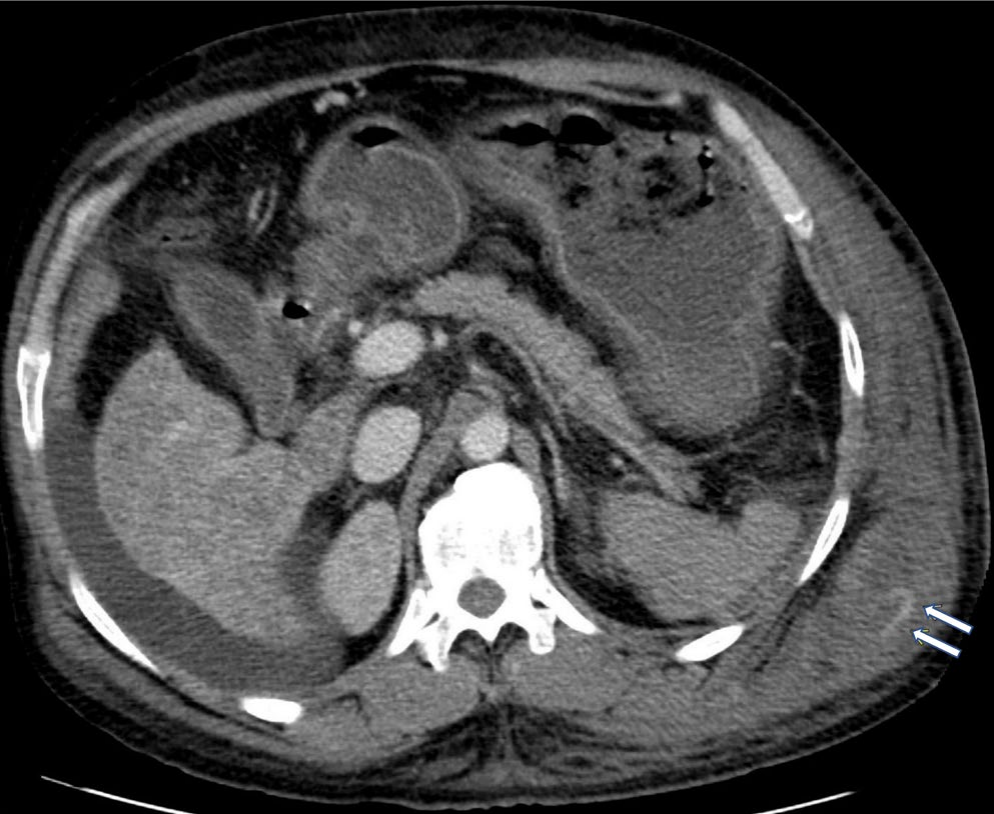
A computed tomography of the chest and abdomen in the axial view showing foci of high attenuation in the intramuscular hematoma involving the left lateral chest wall suggestive of acute bleeding, denoted by white arrows

**FIGURE 3:**
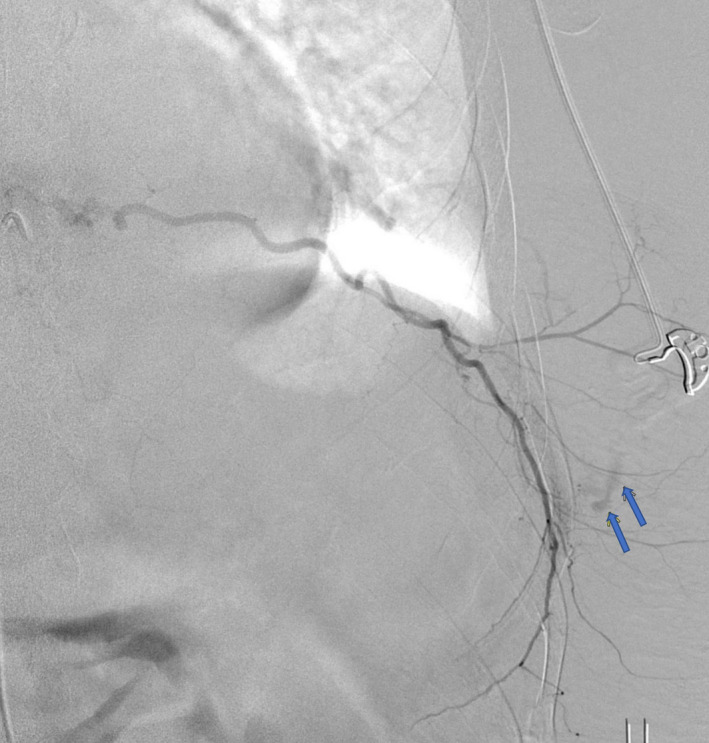
Angiographic image of the left intercostal arteries showing active extravasation from the ninth intercostal artery, denoted by blue arrows

**FIGURE 4:**
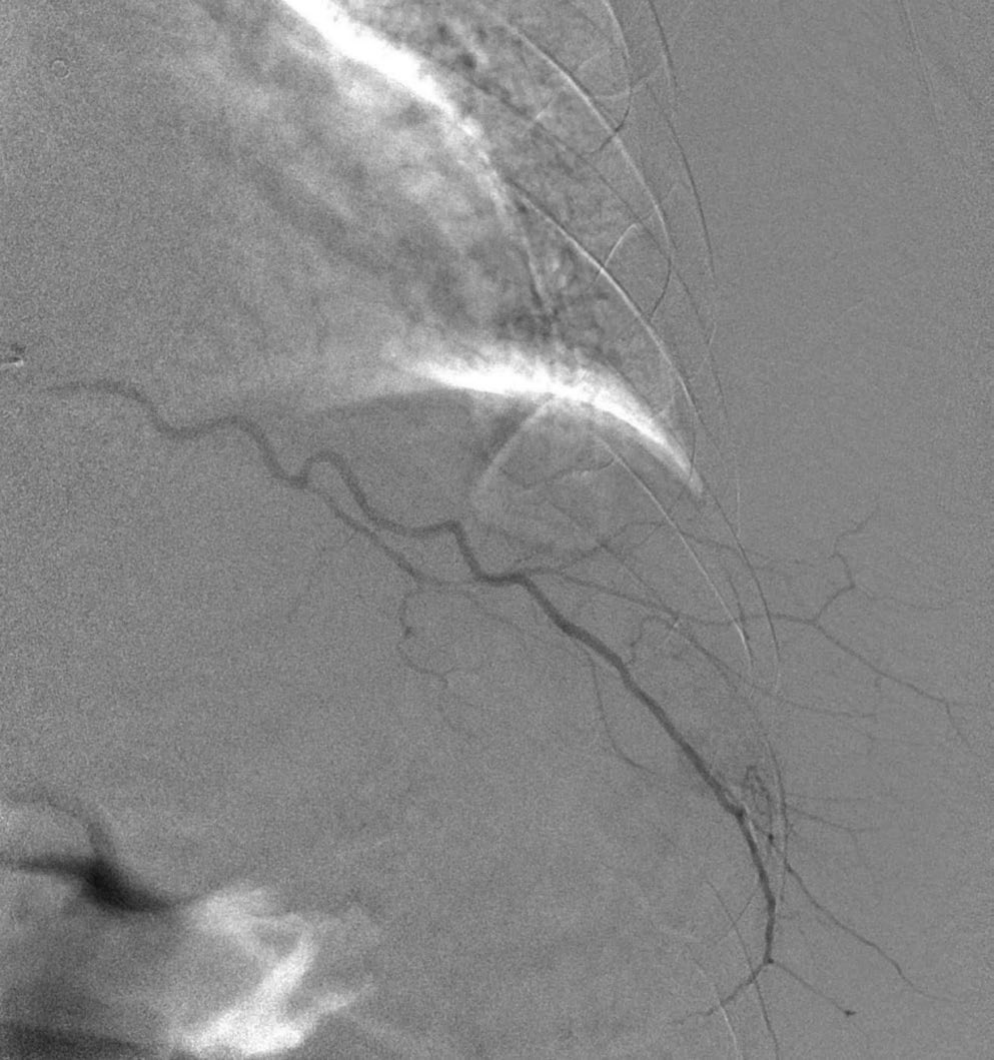
Angiographic image of the left intercostal arteries showing no extravasation after successful embolization of the right intercostal artery

A 44‐year‐old male with alcoholic cirrhosis presented with one day of fatigue. He noticed a painful bruise over his body's left side but denied any trauma or falls. On arrival, he was hypotensive with a hemoglobin of 2.7 g/dl, platelet count of 46,000/mm^3^, and INR of 2.04. Computed tomography of the chest and abdomen revealed a large intramuscular hematoma within the left lateral chest wall extending to the upper abdomen measuring 12 cm × 5 cm and several foci of high attenuation suggestive of acute hemorrhage (Figures 1 and 2). The patient was aggressively resuscitated with our institution's massive transfusion protocol, including four units of red blood cells, four units of fresh frozen plasma (FFP), and one apheresis unit of platelets. Interventional radiology was consulted, and emergent angiography was performed. Active extravasation from the left ninth intercostal artery was noted. Successful embolization with 500‐ to 700‐micron embospheres followed by gel foam slurry was performed (Figures 3 and 4). Spontaneous intercostal artery bleeding is rare in cirrhosis, with few cases reported so far.^1^ Risk factors, in addition to thrombocytopenia and clotting factor deficiency in cirrhotic patients, are alcohol use, trauma, hypertension, and anticoagulation.^2^ Management of closed space spontaneous bleeding in cirrhotic patients is challenging. In mild cases, medical management with judicious use of blood products should suffice. Severe cases are managed with transcatheter arterial embolization and, ultimately, liver transplantation.

## CONFLICT OF INTEREST

None declared.

## AUTHOR CONTRIBUTIONS

All authors made substantial contributions to the conception or design of the work, the acquisition, analysis, or interpretation of data for the work, drafted and assisted in critical revisions to work for important intellectual content, provided final approval of the version to be published, and are in agreement to be accountable for all aspects of the work in ensuring that questions related to the accuracy or integrity of any part of the work are appropriately investigated and resolved.

## ETHICAL APPROVAL

Our study did not require an ethical board approval because it did not contain human or animal trials. Consent was obtained.

## Data Availability

Data sharing not applicable to this article as no datasets were generated or analysed during the current study.
